# Case report and literature review: Isolated HCC- recurrence in gallbladder after curative resection

**DOI:** 10.3389/fsurg.2023.1115181

**Published:** 2023-04-27

**Authors:** Shi-Ran Zhang, Yu Ma, Bo Zhou, Guang-Yao Li, Ping Chen, Geng Chen

**Affiliations:** Department of Hepatobiliary Surgery, Daping Hospital, Army Medical University, Chongqing, China

**Keywords:** liver resection, hepatocellular carcinoma (HCC), recurrence, gallbladder (GB) mass, metastasis

## Abstract

**Background:**

Liver resection (LR) is considered the mainstay treatment for eligible patients with hepatocellular carcinoma (HCC) and provides a 5-year overall survival (OS) of 60%–80%. However, the recurrence rate within five years after LR remains high, ranging from 40% to 70%. Recurrence in gallbladder after liver resection is extremely rare. Here, we present a case of isolated recurrence in gallbladder after curative resection of HCC and review the relevant literature. No similar cases have been reported before.

**Case presentation:**

A 55-year-old male patient was diagnosed with HCC in 2009 and subsequently underwent a right posterior sectionectomy of the liver. In 2015, the patient underwent liver tumor radiofrequency ablation and three transarterial chemoembolization (TACE) procedures in succession for HCC recurrence. In 2019, a gallbladder lesion was detected by computed tomography (CT) without perceivable intrahepatic focus. We performed an *en bloc* resection of the gallbladder and hepatic segment IVb. The pathological biopsy suggested that the gallbladder tumor was moderately differentiated HCC. The patient survived more than 3 years in good condition, and there were no signs of tumor recurrence.

**Conclusions:**

In patients with isolated gallbladder metastasis, if the lesion can be resected *en bloc* without remnants, surgery should be the preferred option. Both postoperative molecularly targeted drugs and immunotherapy are expected to improve the long-term prognosis.

## Introduction

Primary liver cancer ranks sixth in terms of incidence and is the third most common cause of cancer-related mortality worldwide (1). Hepatocellular carcinoma (HCC) accounts for 80% of all forms of liver cancer cases, followed by intrahepatic cholangiocarcinoma (iCCA) (14.9%) and other specified histology (5.1%) (2). HCC is a highly malignant disease (3). Liver resection (LR) is considered the mainstay of curative treatment for eligible patients with HCC and provides a 5-year overall survival (OS) of 60%–80%. However, the recurrence rate within five years after LR remains high, ranging from 40% to 70% (4). It has been reported that intrahepatic recurrence accounts for 66% of relapse cases, while extrahepatic recurrence accounts for 33% (5). Even in advanced HCC tumors, intrahepatic recurrence remains predominant (6–8). Gallbladder metastasis from HCC is a rare clinical occurrence. Autopsy studies report the frequency of gallbladder metastasis as approximately 1.8%–5.8% (9–11). So far, there is still no consensus in the world about the treatment of this rare condition. We underwent a comprehensive review of relevant literature, and only 21 cases of intrahepatic HCC with gallbladder lesions presented synchronously or metachronously have been reported. All of these cases are summarized in Table 1.

**Table 1:** Characteristics of metastatic hepatocellular carcinoma to the gallbladder.

Case	First author	Year	Age/sex	Background	HCC			management	Synchronous	PVTT	Morphologic type	Histological type	Prognosis
					Location	Size(cm)	Number						
1	Terasaki (12)	1990	71/F	Non B non C	S2/3/4	NA	Mt	None	S	+	GBTT/MIG	NA	Soon died
2	Maruo (13)	1994	73/M	Non B non C	S4	4.8	St	Lt. hepatectomy	S	−	Elevated	Mod	32 mo alive
3	Nishida (14)	1997	48/M	HBV	S4/5	NA	Mt	Wedge resection of the gallbladder bed	S	+	Diffuse	Mod	NA
4	Lane (15)	2002	78/M	NA	Right Lobe	NA	Mt	Wedge resection of the gallbladder bed	S	NA	GBTT	Well	Died of pneumonia
5	Chiba (16)	2002	50/M	HCV	S5	5	St	anticancer agents	S	+	GBTT/MIG	Mod	6 mo dead
6	Hwang (17)	2003	65/M	HBV	S4/8	4	Mt	TACE + cholecystectomy	M	NA	Polypoid mass	Poor	NA
7	Terashima (18)	2007	49/M	HBV	S5/6/7/8	10.7	Mt	TAC + cholecystectomy	M	+	Polypoid mass	Mod	13 mo alive
8	Ando (19)	2009	75/M	HCV	NA	NA	Mt	Wedge resection of the gallbladder bed	S	NA	Pedunculated	NA	NA
9	Murakami (20)	2010	53/M	HBV Alc	S7/8	14	Mt	Rt. hepatectomy	S	+	GBTT	Poor	63 mo alive
10			61/M	HCV	S5/8	9.5	Mt	Rt. hepatectomy	S	+	GBTT	Poor	4 mo alive
11			79/M	Alc	S2/3/4	13	Mt	Rt. hepatectomy	S	+	GBTT	Mod	6 mo dead
12			47/M	HBV	S4	6.5	Mt	Rt. hepatectomy	S	+	NA (mp)	Poor	54 mo dead
13			47/M	HBV	S2/3/4	13	Mt	Rt. hepatectomy	S	+	NA (mp)	Poor	9 mo dead
14			32/M	HBV Alc	S5/6/7/8	15	Mt	Rt. hepatectomy	S	+	NA (mp)	Poor	3 mo dead
15			74/M	HCV	S5/6	5	St	Rt. hepatectomy	S	+	NA (sm)	Poor	5 mo dead
16			66/M	Alc	S5/8	3.5	Mt	Rt. anterior sectionectomy	S	+	Protruding (m-mp)	Poor	6 mo dead
17	Monden (21)	2011	66/M	HCV	S5/8/	NA	St	TAC + RFA + cholecystectomy	M	−	Elevated + diffuse	Mod	10 mo alive
18	Kanzaki (22)	2011	48/F	Non B non C	S5	1.3	St	Wedge resection of the gallbladder bed	S	−	Below serosa	Mod	24 mo alive
19	Wakasugi (23)	2012	74/M	HCV	S1/5/6/7/8	8.8	Mt	Wedge resection of the gallbladder bed	S	+	GBTT	Poor	2 mo dead
20	Choi (24)	2012	62/M	NA	S2/4/5/8	NA	Mt	TACE + cholecystectomy	M	NA	Polypoid mass	Poor	NA
21	Hanazawa (25)	2021	66/F	Non B non C	S5/6/7/8	12	St	Rt. trisectionectomy	S	+	MIG	Poor	42 mo alive
22	Present case	2022	55/M	Non B non C	S4/7/8	NA	Mt	TACE + Wedge resection of the gallbladder bed	M	−	MIG	Mod	40 mo alive

The vast majority of cases were found at the time of routine concomitant gallbladder pathological examination after hepatectomy. Gallbladder lesions were found in only 4 cases during post-treatment follow-up after the primary liver tumors had been managed for 1–3 years. The initial treatment methods in all of these 4 cases are TACE and/or RFA. Since no case of isolated gallbladder recurrence after curative resection of HCC has been reported to date, we document this extremely rare case.

## Case presentation

**Figure 1:**
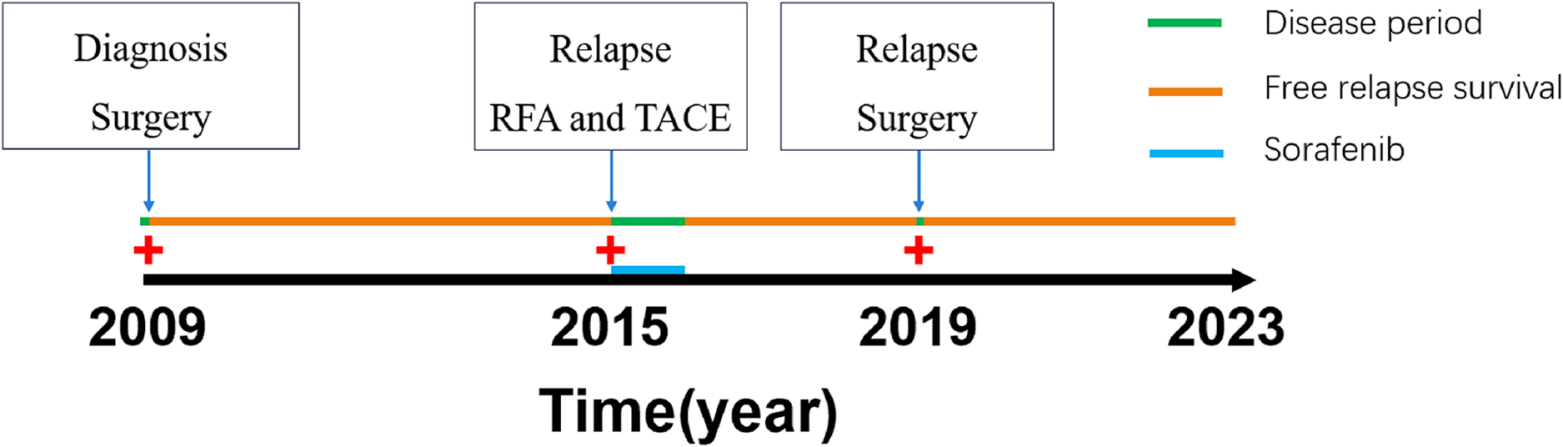
The timeline of patient's history.

**Figure 2:**
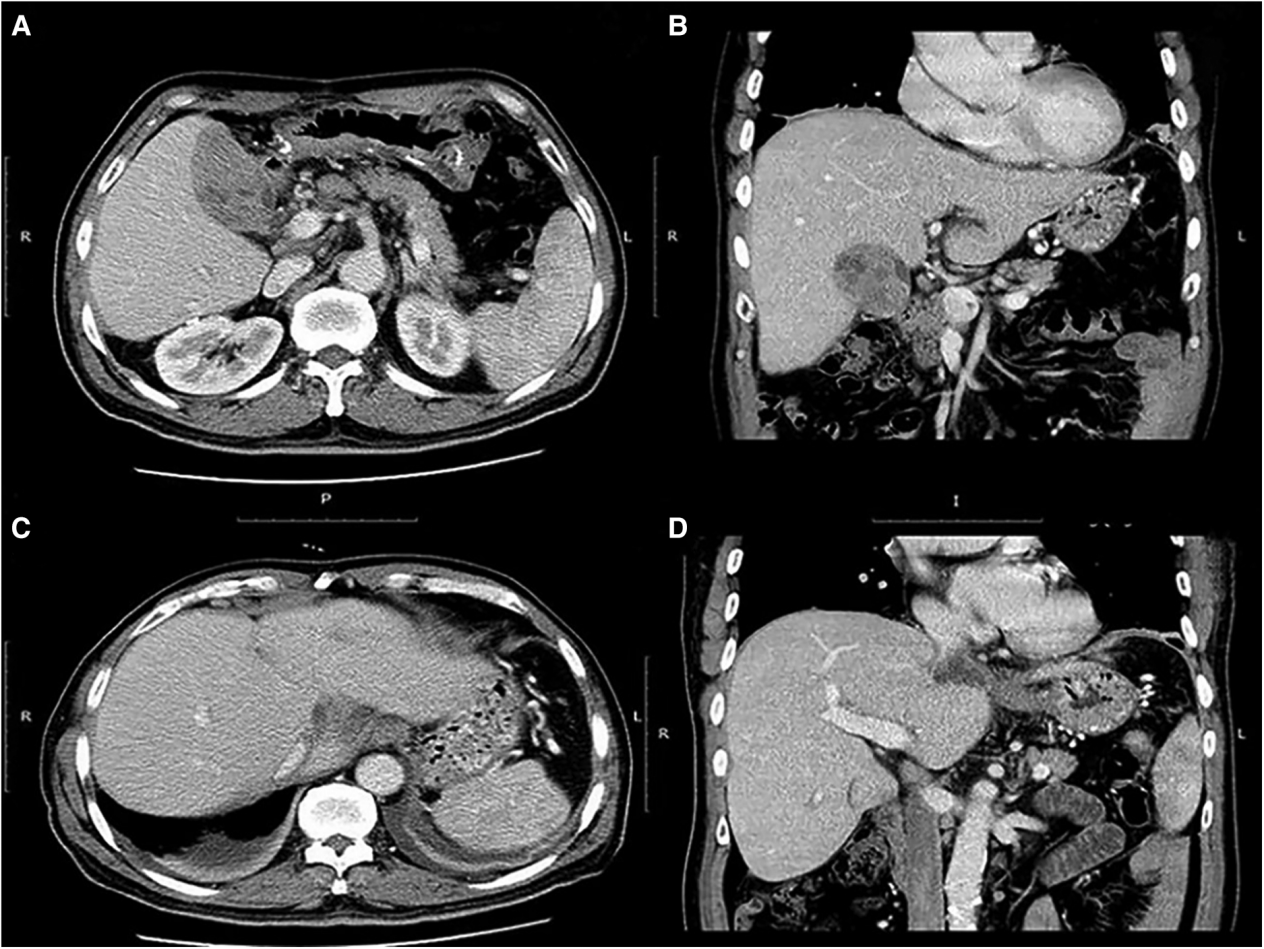
(**A**) computed tomography (CT) depicted a gallbladder tumor with dimensions of 8 × 4 cm. (**B**) Coronal image shows a clear demarcation between the tumor and the adjacent liver parenchyma. (**C**) No perceivable intrahepatic foci were found. (**D**) The right portal vein is tumor thrombus free.

In our case report, a 55-year-old male patient was admitted to our department for recurrent pain in the upper-middle abdomen, which had started 2 years earlier and was aggravated with jaundice for 3 days. The patient complained of pain and discomfort that often radiated to the back and was accompanied by nausea and vomiting. Three days before admission, the pain flared up again, chills and fever were accompanied, and jaundice followed. The patient's prior medical history is as follows. In 2009, he received right posterior sectionectomy for primary liver cancer. In 2015, he received TACE and radiofrequency ablation for the treatment of recurrent HCC. In the meantime he took Sorafenib for one year. (Figure 1). Physical examination showed that the patient had skin and scleral jaundice and tenderness in the right upper quadrant. Laboratory tests indicated the following: white blood cell count (WBC) 16.47 × 10^9^/L, neutrophil ratio (NEUR) 94.9%; albumin (ALB) 37.1 g/L, total bilirubin (TB) 125.7 μmol/L, direct bilirubin (DB) 72.9 μmol/L, aspartate aminotransferase (AST) 111.5 U/L, alanine transferase (ALT) 355.8 U/L, gamma-glutamyltransferase (GGT) 289.1 U/L; serum α-fetoprotein (AFP) 401.23 ng/ml; prothrombin time (PT) 18.8 s, prothrombin time international normalized ratio (PT-INR) 1.64; procalcitonin (PCT) 4.77 ng/ml, and interleukin-6 (IL-6) 201.5 pg/ml. Hepatitis B surface antigen and hepatitis C virus antibody were negative. An abdominal computed tomography (CT) scan showed that the lesion filled the gallbladder cavity. The wall of the lower segment of the common bile duct was thickened. Liver cirrhosis was also revealed. This gallbladder tumor showed punctate enhancement in the hepatic arterial phase and then became less dense than the liver parenchyma in the portal phase (Figure 2). No regional lymph nodes were enlarged.

**Figure 3:**
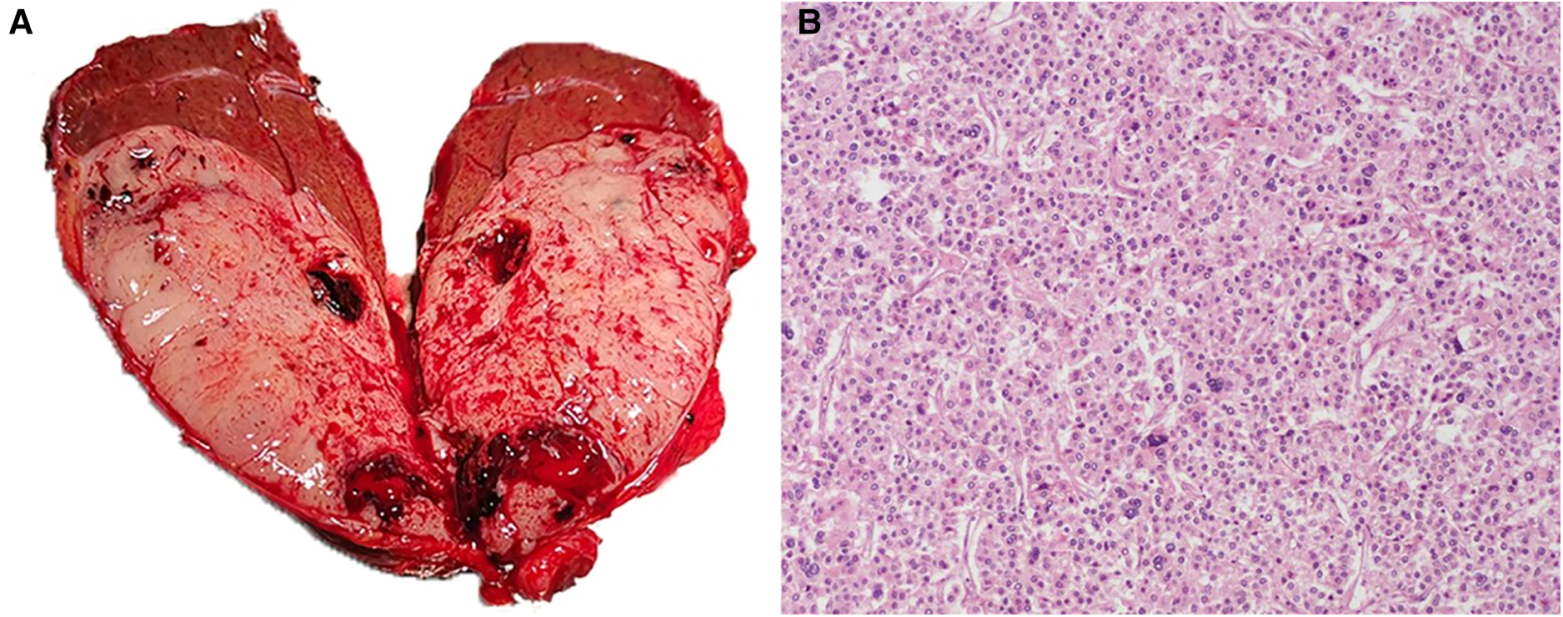
(**A**) macroscopic examination shows that the gallbladder lumen is filled with white fish-like tissue. (**B**) Histopathological examination revealed moderately differentiated hepatocellular carcinoma cells (hematoxylin and eosin, × 100).

The preoperative diagnosis was considered gallbladder tumor with acute cholangitis, which was potentially malignant. Therefore, hepatectomy of segment IVb, cholecystectomy and common bile duct exploration were planned in this case. Intraoperative exploration revealed extensive intraperitoneal adhesions and gallbladder enlargement with dimensions of 12 cm × 4 cm × 3 cm. The gallbladder was solid and tough, while the liver was soft. Because there was no metastasis to the lymph nodes or peritoneum, we performed a monobloc resection of the gallbladder and hepatic segment IVb, and common biliary duct exploration was also performed. The frozen section of the intraoperative specimen indicated a malignant tumor (gallbladder lumen occupying) and probable metastasis of HCC. Subsequent biliary exploration found several stones in the lower part of the common bile duct. After the stones were removed, the lower part of the common bile duct was observed with a choledochoscope, and no tumor invasion or neoplasm was found. Macroscopically the diseased gallbladder appeared to be filled with white soft tumor tissue, and some tissue had extended along the cystic duct to the common bile duct (Figure 3A). The operative time was 360 min, and blood loss was 200 ml. The postoperative paraffin histopathology and immunohistochemistry showed a moderately differentiated HCC with necrosis (Figure 3B). The final diagnosis was amended to HCC recurrence in gallbladder, choledocholithiasis and acute cholangitis. Antibiotics and hepatoprotective drugs were used in postoperative management, and the patient recovered uneventfully within 10 days.

The follow-up showed that the patient was in good condition and survived more than 40 months with no signs of tumor recurrence. To date, the patient has not taken any targeted drugs and immunotherapy drugs we recommended for treatment after surgery. The patient refused the medication because of the significant side effects of sorafenib at the time of the first relapse.

HBV, hepatitis B virus; HCV, hepatitis C virus; Alc, alcoholic hepatitis; Mt, multiple tumors; St, single tumor; NA, not available; S, synchronous; M, metachronous; GBTT, gallbladder vein tumor thrombus; MIG, massive intragallbladder growth; mp, muscularis propria; m-mp, mucosal layer-muscle layer; sm, submucosal layer; Mod, moderate; mo, month.

## Discussion

As the tributaries of the portal vein are vulnerable to the invasion of HCC, the prevalence of portal vein tumor thrombosis (PVTT) is widespread in advanced HCC patients. HCC cells can be shed from the original PVTT by the portal vein blood flow and disseminate to the distal or even proximal perfusion areas. Therefore, intrahepatic metastasis occurs frequently. In terms of gallbladder metastasis from HCC, Nakashima et al. proposed four possible pathways: (1) hematogenous metastasis through the portal venous system; (2) lymphatic metastasis; (3) direct invasion from adjacent liver parenchyma; and (4) peritoneal dissemination (11). The first pathway was considered the most likely route of HCC cell migration to the gallbladder. In patients with liver cirrhosis, the portal vein flow velocity is reduced compared with that of normal liver (26). Meanwhile, the incidence of bidirectional and reversed flow in the portal venous system in cirrhosis is 10.8% (27). Portal flow can even be occluded by occupation of the entire lumen by PVTT. Sugita et al. identified 72 cystic veins in 27 patients in their research and revealed that all cystic veins drained into the intrahepatic sinusoids or portal branches *via* the hepatic hilum (17 patients, 21 veins) and hepatic bed (23 patients, 51 veins) (28). The portal branches and sinusoids in subsegment 4b and segment 5 are usually involved in the drainage of the cystic vein. Under these conditions, retrograde movement of HCC cells is possible, which may result in tumor invasion along the gallbladder vein into the gallbladder lumen. The above anatomic features of the cystic vein coincide with the occurrence site of HCC in the liver. In 19 out of 22 cases, the HCC tumor was located in segment 4 and/or segment 5, one case's location was not available, and one case was located in segment 7/8. PVTT was encountered in 14 cases. Histopathological examination confirmed the presence of gallbladder vein tumor thrombosis (GBTT) in 7 cases. Murakami et al. defined “metastasis” through the cystic vein as “local extension”, which is a more appropriate description of this hematogenous route of gallbladder spread (20).

In terms of this particular case, preoperative differential diagnosis of primary gallbladder carcinoma and gallbladder metastasis from HCC is difficult due to the lack of typical imaging features. HCC only rarely breaks through the muscle layer and collagen fibers of the gallbladder wall, whereas gallbladder carcinoma can easily infiltrate the liver (16). All cases we reviewed were confirmed as metastasis by postoperative histopathological examination of the resected specimen. This patient's liver cancer first arose in the right posterior lobe of the liver in 2009, which is not close to the gallbladder bed, but recurrence occurred several times after surgery with tumors located in segment 4. HCC cells may migrate from recurrent lesions through the branches or sinusoids between the cystic vein and the portal vein. The pathological features were also similar to those of several other metachronous cases. Metachronous gallbladder tumors are mainly characterized by polypoid growth in the gallbladder lumen and are often detected by CT, whereas synchronous gallbladder tumors are usually found by postoperative pathological examination and are often characterized by the presence of gallbladder vein tumor thrombus (GBTT). Once extrahepatic metastasis of liver cancer occurs, the prognosis of surgical resection is often poor. Uchino et al. reported that the 1-year survival rate was 39.3%, and the 3-year and 5-year survival rates were 7.4% and 4%, respectively (8).

However, we noticed that the recurrent tumor in this case mainly grew in the gallbladder lumen and entered the common bile duct, causing biliary obstruction instead of invading the gallbladder muscular layer and serosa. The same phenomenon was also found in other cases. This indicates that recurrent gallbladder tumors are more inclined to follow the growth pattern of primary hepatocellular cancer, such as growing toward areas of lower pressure, including veins and bile ducts, to form a tumor thrombus. Primary gallbladder cancer, meanwhile, tends to grow more aggressively by infiltrating the gallbladder wall (16).

Secondary HCC is often classified as multicentric carcinogenesis (MC), intrahepatic metastasis (IM), and extrahepatic metastasis. Previous studies have suggested that early recurrences (≤1 year after primary lesion resection) appear to arise mainly from IM, whereas late recurrences (>1 year after primary lesion resection) are more likely to be the result of MC (29). Arii et al. showed that patients with extrahepatic metastasis originating from MC had a significantly better outcome than those originating from IM (29). In the current study, the patient's primary lesion that appeared in 2009 was surgically removed. Multiple intrahepatic recurrences that occurred in 2013 were cured with local treatment. As the patient had been disease free for 4 years prior to recurrence, the recurrences can be considered the result of MC. In terms of extrahepatic metastases, Yang et al. divided extrahepatic metastases after liver resection into three types. Pattern I: first recurrence in the liver followed by spread outside the liver after repetitive intrahepatic recurrences and repetitive locoregional treatments, pattern II: intrahepatic and extrahepatic recurrences exist simultaneously, and pattern III: extrahepatic recurrence without intrahepatic lesions at first recurrence (30). According to their study, pattern I was significantly better than pattern II in terms of overall survival and disease-free survival rates. This case corresponds exactly to pattern 1, with repetitive intrahepatic recurrences after resection of the primary liver tumor and finally development of isolated gallbladder metastasis. After wedge resection of the gallbladder bed and administration of an anticancer agent, the patient finally had a good prognosis.

There is no consensus on the management of extrahepatic metastasis. Surgical resection and antitumor agents are currently predominant in management. According to Murakami et al. (20) and Hanazawa et al. (25), favorable results were obtained using adjuvant therapy after resection of recurrent gallbladder tumors from HCC. This indicates that the outcomes of recurrent HCC in gallbladder undergoing radical resection are better than those of primary gallbladder cancer. Therefore, for patients with isolated recurrent gallbladder tumors, if the lesion can be resected *en bloc* without remnants, surgical resection should be performed aggressively. Both postoperative molecularly targeted drugs and immunotherapy are expected to improve the survival rate and prognosis of patients.

## Data Availability

The original contributions presented in the study are included in the article/Supplementary Material, further inquiries can be directed to the corresponding author.
